# Adaptation of the TIMBRE methodology for brownfields gully erosion analysis in urban areas

**DOI:** 10.1016/j.heliyon.2024.e32902

**Published:** 2024-06-12

**Authors:** Caiubi Emanuel Souza Kuhn, Fábio Augusto Gomes Vieira Reis, Flávia Regina Pereira Santos, Christiane Zarfl, Peter Grathwohl, Victor Cabral

**Affiliations:** a Universidade Estadual Paulista Júlio de Mesquita Filho (UNESP), Av. 24 A, 1515, 13506-692, Bela Vista, Rio Claro, Estado de São Paulo, Brazil; b Universidade Federal de Mato Grosso, R. Quarenta e Nove, 2367 - Boa Esperança, 78060-900, Cuiabá, Estado do Mato Grosso, Brazil; c Geo-Umweltforschungszentrum (GUZ), University of Tuebingen, Schnarrenbergstr. 94-96, 72076, Tubingen, Germany

**Keywords:** Greenfields, Brownfields regeneration, Soil degradation, Gully remediation

## Abstract

Soil erosion is a concern in many parts of the world, causing environmental and social impacts. Aiming at obtaining indicators of the recovery of brownfields created by gullies in urban areas, this study adapts the Tailored Improvement of Brownfield Regeneration in Europe (TIMBRE) for the analysis and classification of areas affected by gullies in the city of Bauru, Brazil. The TIMBRE methodology assists in the decision-making of priority areas for remediation and their reinsertion in urban spaces. The inventory of areas affected by gullies was compiled based on the analysis of two image sets (2004 and 2020) available on Google Earth. For the classification of brownfields, three classes were considered: Class 1 - local potential for business development, Class 2 - attractiveness and marketing, and Class 3 – environmental risks. These results demonstrate a correlation between gullies and urban expansion. The inventory identified 175 gullies in the municipality's urban perimeter in 2004, which affected an area of over 64 ha. In 2020, the number of gullies increased to 189, but the affected area decreased to 62 ha due to the recovery of some large gullies. The proposed methodology identified the area of Quinta da Bela Olinda as the one with the highest scores in all three classifications. Quinta da Bela Olinda is the location that has a local potential for business development, as it is the most attractive brownfield, as well as the area with the highest environmental risk. This area should, thus, be prioritized by public management for remediation. In conclusion, the proposed method of analysis can be transferred to other areas with adaptations in the criteria used and, therefore, may facilitate the management of urban areas affected by gullies in other places around the world.

## Highlights

• Gullies can be considered a form of brownfields.
• The TIMBRE methodology can be adapted for gully remediation.
• Remediating gullies are important to improve urban management.
• Brownfield analysis is needed to improve land use.

## 1 Introduction

Gully erosion is a global concern of soil deterioration; it limits land use, affects infrastructure and, in cities, it creates urban voids/brownfields [[1], [2], [3]]. The impact of gullies on economic and ecosystem services are most commonly registered in Brazil, India, China, the Democratic Republic of Congo, Ethiopia and the United States, but damage also occurs in countries such as Algeria, Argentina, Australia, Iran, Italy, Mexico, Nigeria, Polonia, Portugal, Romania, Russia, Slovakia, South Africa, Spain, Swaziland, Tanzania, Tunisia, Turkey, Ukraine and the United Kingdom [4].

Although studies that address the development of gullies in rural areas are common [[5], [6], [7]], only 3.1 % of published studies about gullies investigated gully erosion in urban areas [5]. Gullies are recorded in hundreds of cities in Brazil, in some cases causing significant damage to urban structures [8]. The issue of gullies in urban space was also the object of studies in Nigeria [9], in Kinshasa, and in the Democratic Republic of Congo [10], among other countries [2,3,5]. In addition to the impacts caused on urban structures, gullies affect significant areas, making the use of these urban spaces unfeasible. Areas affected by gullies can be restored, but if the necessary interventions are not enacted, they become urban voids [1,11].

Although factors such as relief, climate, extreme rainfall events, geology, and soil type can influence susceptibility to the development of gullies, there is a consensus in the literature that human action or extreme events are important triggers for their development [3,5,12,13]. Gully control results in various positive effects, such as on water flow regulation and biological diversity, and it reduces siltation and the risk of infrastructure damage [14].

In cities where there are hundreds of gullies, especially in developing countries, where financial resources are scarce [4,[9], [10], [11]], creating criteria to know which erosions should be prioritized for remediation and reinsertion of the area into the urban environment becomes a necessary action. This study presents contributions to ranking erosion to assist in decision making, using as a basis methods developed for brownfield analysis.

Gullies in urban spaces create problems in territorial and environmental management comparable to brownfields [11]. Brownfields [15] are "places that have been affected by previous uses of the surrounding place or land; they are abandoned or underused areas that are mainly in totally or partially developed urban spaces, requiring intervention to bring them back to beneficial use and may have real or perceived contamination problems”. The areas affected by gullies also had a previous use, and after the triggering event, the area is underused or abandoned, requiring intervention to remediate the area and, when possible, return it to beneficial use [4,8,11]. Brownfields are not necessarily contaminated areas, they can be considered areas with previous use, which have not been reintroduced for a new one [16,17]. Thus, in cities heavily affected by gullies [[8], [9], [10]], this process can be considered as a form of brownfield.

Brownfield rehabilitation is a topic that has attracted the attention of researchers in recent decades in countries such as the United States, England, Canada, Germany, China, Italy, Czech Republic, Spain and Australia [18]. Decision support tools are potentially effective for brownfield evaluation and for the development of strategies for remediation or management of affected areas [19].

Recovery of brownfields preserves the environment. or the so-called Greenfields, which provide important ecosystem services [20]. However, brownfield recovery requires significant resource allocation in terms of time and cost, which often exceeds the cost of greenfield development [21].

The Brownfield Prioritization Tool “TIMBRE” is a decision-supporting methodology created for Brownfields analysis considering the economic, social and environmental dimensions [21,22]. Brownfield analysis is performed in three steps [23]: 1) Inventory (mapping, problem identification and analysis), 2) Prioritization (assessment of redevelopment potential and environmental risks), and 3) Marketing (fundraising and investor search).

This work aims at applying a multiple criteria analysis based on the adaptation of the criteria of the Brownfield Prioritization Tool “TIMBRE”,for the analysis of areas affected by gullies. The city of Bauru in Brazil is selected as our test-site, due to the impacts that gullies have on urban structures. We hypothesize that the TIMBRE methodology allows quick responses to gully management.

## 2 Method

**Fig. 1:**
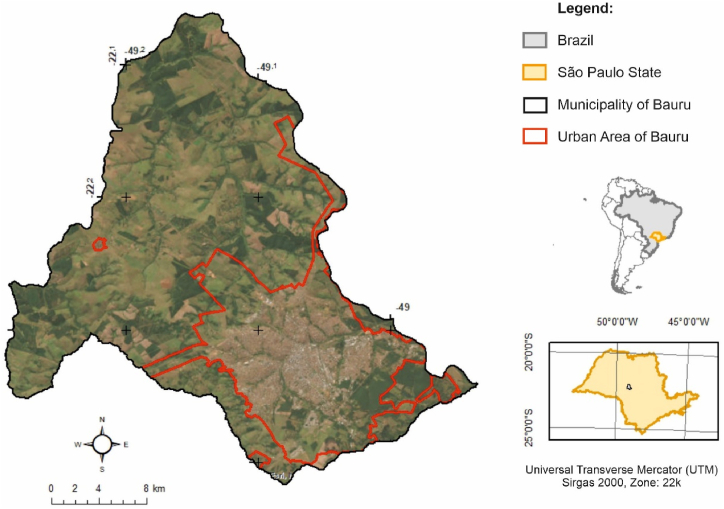
Location of the study area (red: urban perimeter of Bauru). (For interpretation of the references to colour in this figure legend, the reader is referred to the Web version of this article.)

The TIMBRE methodology proposes different levels of decision hierarchies to analyse brownfields [23], which we adapted for the analysis of gully-affected areas. In the adaptation proposed, three stages are used: a) Inventory - in which the gullies are classified according to their activity level; b) Prioritization - three classes are used, based on the sum of the scores of the proposed parameters described in section 2.2; c) Marketing - where the results are integrated to identify and discuss priority areas for recovery. The urban perimeter of the city of Bauru was chosen for the case study (Fig. 1).

### 2.1 Inventory

The inventory step includes the mapping, identification and analysis of gullies. The identification of gully scars was performed using images from Google Earth from 2004 to 2020, extracting the location and size of the area affected on the two dates analysed. The use of aerial images for gullies analysis is a common method in many studies [[24], [25], [26], [27]]. Gullies were classified into three classes: a) “recovered areas”, which represents areas that had their function in relation to land use recovered between 2004 and 2020 (e.g., they were filled with materials, aiming at the construction of houses, streets, urban parks, among others); b) “stable areas”, representing where no further evolution of gullies in the considered period was visible, such as areas with consolidated vegetation (trees or grass) that do not show signs of sediment removal (e.g., silted areas); and c) “unstable”, where gullies progressed visibly in satellite images (vegetation not consolidated and siltation occurred in channels or in downstream locations).

### 2.2 Prioritization

Prioritization was based on the analysis of land use and occupation according to Bauru's Master Plan [28]. Thematic maps were used to obtain information on urban infrastructure, population density, education and sanitation infrastructure, characteristics of soil use, transport structures, health services, development areas for new enterprises and proximity to watercourses. For development of the index of risks, gullies magnitude and activity, the results from the inventory stage were used. Land value was calculated based on the territorial taxes charged per square metre by the municipality [29]. The proximity of gullies to built areas was defined based on a map composition of spectral bands produced with the use of Landsat 8 orbital images extracted from the Portal of the National Institute for Space Research (INPE). The set of data used must be suitable for the municipal scale; i.e., to provide an analysis on the scale used by municipal managers to make decisions on urban planning.

The classification maps were created with arithmetic functions in geoprocessing software, so that the parameter scores could be established. The functions used were union, intersection and subtraction of polygons from the shapefiles of the Master Plan database provided by Bauru City Hall [29] or development of contour maps, created using the Inverse Distance Weighting interpolation method to establish the spatial variation of the value of the characteristic. The gullies were identified and georeferenced in a shapefile of points due to the adopted scale, which were classified by attributes, and later intersected with the areas of the municipality. The priority categories were defined as follows: 1) Class 1: “local potential for business development” comprising land value, population density, educational index, entrepreneurial activity, periphery and transportation connections (Table 1); 2) Class 2: “attractiveness and marketing potential” considering specific location, previous use, infrastructure, and expected regeneration costs (Table 2); and 3) Class 3: “environmental risks” accounting for gullies magnitude, activity (stable or dynamic) of the erosive process, location with respect to urban zonation and proximity to built areas (Table 3). The priority ranking was evaluated on a scale score from 0 to 4. Three Table 1, Table 2, Table 3 summarize the methods of the prioritization.

**Table 1:** Classification of “Local potential for business development”.

Parameter	Method for calculation	Score	Description
Land value per square metre	land value per square metre provided by the municipality of Bauru	0	Properties located in rural or urban area with value of up to US $ 6 per square metre
		1	Properties located in urbanized or periurban regions, in neighbourhoods with low to medium standards and values between $ 6.1 and $ 60 per square metre
		2	Properties located in urbanized regions with values between $ 60.1 and $ 160 per square metre.
		3	Properties located in urbanized regions, in neighbourhoods with medium to high standards with values of over US $ 160.1 per square metre.
Population density	Heatmap on housing density	0	Up to 500 inhabitants per km^2^
		1	Between 501 and 2500 inhabitants per km^2^
		2	Between 2501 and 5000 inhabitants per km^2^
		3	Above 5001 inhabitants per km^2^
Educational indices	Distance to an educational unit (schools and university)	1	Areas without availability of educational units within a radius of up to 1.5 km
		2	Areas with one educational unit within a radius of 1.5 km
		3	Areas with two or more educational units up to 1.5 km distance
Sanitation	Distance from road with networks of water and sewage collection	1	Area without sewage and water and more than 200 m away from the municipal road network
		2	Area without sewage and/or water and far less than 200 m from the municipal road network
		3	Area with complete sanitation and water structure within the municipal road network
Land use	Use of the areas indicated in the Master Plan	1	Non-urbanized area
		2	Area in urbanization, urban voids and green area
		3	Areas located in urbanized regions.
Transportation connections	Distance from the main access roads	1	More than 100 m from some street and over 500 m from avenues and highways.
		2	Less than 100 m from some street or less than 500 m of avenues and highways.
		3	Less than 100 m from some street and less than 500 m from avenues and highways.
Health structures	Heatmap considering the distance from health facilities	1	Areas without availability of health units within a radius of up to 1.5 km.
		2	Areas with low concentration of health units within a radius of 1.5 km
		3	Areas with high availability of health units within a 1.5 km-distance

“Local potential for business development” was quantified by summing the scores attributed to each parameter (e.g., Land value per square metre + Population Density + Educational Indices + Sanitation + Land use + Transportation connections + Health Structure).

**Table 2:** Attractiveness and marketing potential.

Parameter	Method for calculation	Score	Description
Infrastructure	Calculated according to the availability of paved roads, water and sewage network, distance from schools and health units	1	No sanitation structure, paved roads, education network and consolidated health network.
		2	With sanitation structure, paved roads, education network, health network, water in consolidation.
		3	With sanitation structure, paved roads, education network, health network, available water.
Potential of future residential use	Calculated as used in the Municipal Master Plan, considering the possibilities of use for homes.	0	Areas not indicated for residential use
		1	Areas indicated for single -family residential use
		2	Areas indicated for horizontal or/and single -family multifamily use
		3	Areas indicated for single -family, horizontal and vertical multifamily residential use.
Possibilities of future uses for trade	Calculated as used in the Municipal Master Plan, considering the possibilities of use for trade	0	Areas not indicated for trade use
		1	Areas where compatible or tolerable activities can be developed in residential areas (e.g. residential galleries, hotels, retailers)
		2	Areas where compatible and tolerable activities occur in residential areas (e.g. retail trade, gas stations, bank branches)
		3	Areas where compatible, tolerable and uncomfortable activities occur in residential areas (e.g. carriers, wholesale trades of fuel storage.)
Possibilities of future uses for industry	Calculated as used in the Municipal Master Plan, considering the possibilities of use for the industry.	0	Areas not indicated for industrial use
		1	Areas where industrial activities can be developed in accordance to residential areas (e.g. manufacturing of food products)
		2	Areas where compatible and/or tolerable industrial activities can be developed by the residential area (e.g. manufacturing of wood products).
		3	Areas where compatible, tolerable and/or uncomfortable industrial activities can be developed to the residential areas (e.g. cosmetics manufacture and cleaning products)
		4	Areas where compatible, tolerable, uncomfortable and incompatible industrial activities can be developed to the residential areas (e.g. metallurgy, slaughterhouses and refrigerators)
Expected regeneration costs	Calculated according to the size of the area of each scar of gully.	1	Gully with an area larger than 10000 m^2^
		2	Gully with area between 2001 and 10000 m^2^
		3	Gully less than 2000 m^2^
Ease of developing new enterprises	Calculated considering restrictions on use and the size of the surrounding areas. The larger the unused area the better the score, because the cost of giving recovery is more easily diluted in areas that can be used by large enterprises.	0	Green area or special use where the building of houses, installation of industries or commerce is not allowed
		1	Consolidated urban area
		2	Allotment with low occupation, controlled occupation zone or areas in river valleys located in consolidated Urban Area
		3	Areas where urban voids predominate
Drainage	Consider whether Gully is in a natural drainage area. There are restrictions on Brazilian law for the development of enterprises in areas of drainage or source.	0	Gully positioned in the valley bottom with natural drainage
		2	Gully positioned in the slope

“Attractiveness and marketing potential” was quantified by summing up the scores attributed to parameter infrastructure, expected regeneration costs, ease of developing new ventures and drainage. The possibilities of future uses can be considered according to municipal planning (residence or commercial or induction).

**Table 3:** Environmental risks.

Parameter	Method for calculation	Score	Description
Gully area size	The higher the size of the gully the higher the risk	1	Gully with an area of up to 2000 m^2^
		2	Gully with an area Gully area size of between 2.001 and 10,000 m^2^
		3	Gully with an area larger than 10,001 m^2^
Erosive Process activity	Score according to stability of the area of Gully.	2	Stable or Gullies
		3	Gullies unstable
Zoning	Calculated according to the proximity to urban infrastructure, being considered the highest grade for areas without structure and close to the urban perimeter.	1	Urbanized area, with control of rainwater surface runoff (zones that are not green areas, built areas).
		2	Not urbanized area with rural use.
		3	Periurban area with low urbanization or green areas within the urban perimeter, and with linear structures that facilitate the concentration of surface water.
Risk	Calculated according to the proximity of the gullies of built areas, based on the analysis of orbital image compositions.	1	Area without residences or public infrastructures over 301 from the surroundings of gully.
		2	Area with residences or public infrastructures between 300 and 101 m from gully.
		3	Area with residences and public infrastructures less than 100 m

“Environmental risk” was quantified by summing up the scores attributed to the parameters (gully area size + Erosive Process Activity + Zoning + Risk), considering the sum of all assigned values.

### 2.3 Marketing

Marketing potential was assessed based on the integration of data from the previous steps, aiming at obtaining indications for future use, as well as identifying trends in relation to land use and occupation in the municipality of Bauru.

In the “attractiveness and marketing class”, the score used for final classification considered the infrastructure, expected recovery (or restauration) costs, connection to drainage and ease of developing new enterprises. The score regarding the possibilities of future, commercial, residential and industrial uses was not considered because each area of the city has specific restrictions for use, as indicated in the Municipal Master Plan for each neighbourhood in the municipality; in this way, only the sum of the other parameters was considered, they being: expected regeneration costs, ease of developing new enterprises, drainage, and infrastructure.

## 3 Results

### 3.1 Inventory

**Fig. 2:**
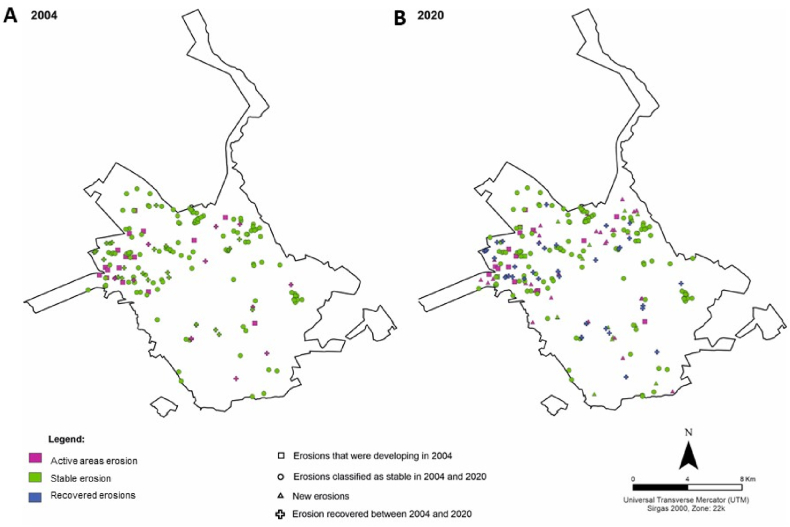
Gullies inventory in 2004 (A) and 2020 (B).

The inventory of gullies demonstrated that in 2004 (Fig. 2 A), 175 gullies existed in the urban area occupying an area of more than 64.1 ha. In 2020 (Fig. 2 B), the number of gullies had increased to 189, with an affected area of 62.5 ha (Table 4).

**Table 4:** Number of gullies and affected area in 2004 and 2020. Stable areas, where no further evolution of gullies during the analysed time interval; and unstable where erosion progressed visibly in satellite images.

Classification		Gullies number and percentages of total		Affected area m^2^ percentages of total	
Gullies in 2004	stable	145	82,9 %	472425	73,6 %
	unstable	30	17,1 %	169043	26,4 %
Total in 2004		175		641468	
Gullies in 2020	stable	139	73,5 %	466400	74,5 %
	unstable	50	26,5 %	159527	25,5 %
Total in 2020		189		625927	

**Fig. 3:**
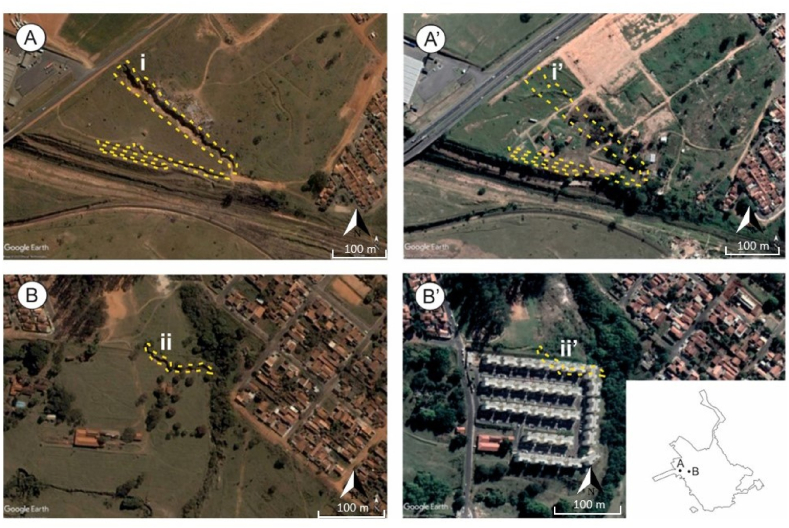
(A) large gully (i) active in 2004; (A′) after remediation channel covered (i’) and reintroduction of the area in the urban environment; (B) gully channel (ii) connected to a watercourse in 2004; (B″) area with social use recovered in 2020, after remediation of the area affected by gullies and the construction of residential buildings (ii’).

Between 2004 and 2020, a total of 37 gullies were mitigated and reintroduced into urbanization with an area of 6.2 ha (Table 5). Most recovered gullies were already stable with vegetation inside the gully channel. However, the area affected by the 13 active gullies that were recovered represented approximately 2/3 of the recovering area (Fig. 3 A, B). Large areas affected by gullies were recovered during this period, generally for residential use or for the construction of urban infrastructure.

**Table 5:** Areas where gullies were recovered between 2004 and 2020.

Gullies recovered between 2004 and 2020	Number and percentages		Affected area m^2^ percentages of total	
Classified as “stable” in 2004	24	64.9 %	21420	34.1 %
Classified as “unstable” 2004	13	35.1 %	41410	65.9 %
Total	37		62830

**Fig. 4:**
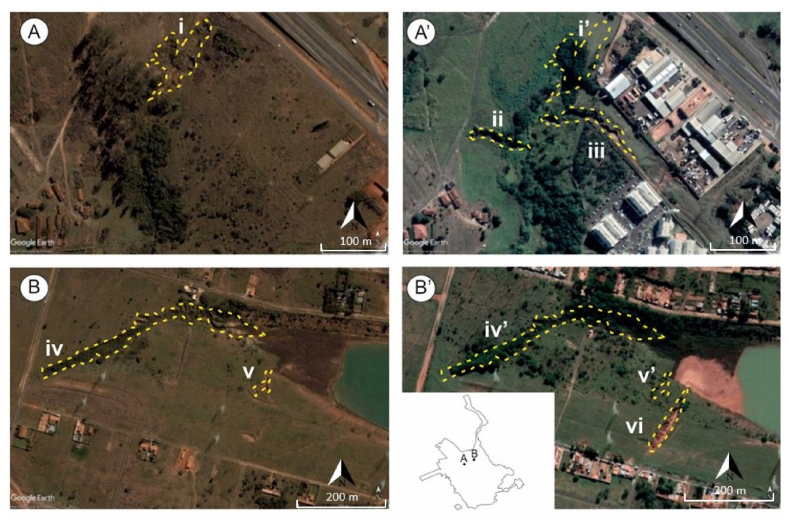
Some areas affected by new gullies between 2004 and 2020; (A) area with stable gully (i) in 2004; (A′) Gully channel development near residential condominium (iii) in 2020; (B) Quinta da Bela Olinda: area with two (iv and v) stable gullies in 2004; (B′) Quinta da Bela Olinda in 2020: area with two stable gullies (iv’ and v’) and a newly developed gully (vi).

Between 2004 and 2020, 51 new gullies (Fig. 4 A, B) were identified, most of which were already active in 2020. However, the number of new gullies is greater than the number of recovered gullies. The new gullies are smaller than the gullies that were recovered, which contributed to the reduction in the area affected by them. Most gullies that were not stabilized in 2004 showed changes in channel development in 2020. The gully-affected area in 2020 is lower than in 2004, but the number of existing gullies is higher (Table 6).

**Table 6:** Existing gullies in the study area in 2020.

Classification		Gully number and percentages of total		Affected area m^2^ percentages of total	
Gullies that emerged between 2004 and 2020	stable	16	8.5 %	11,077	1.8 %
	unstable	35	18.5 %	33,265	5.3 %
Gullies classified as stable in 2004 and 2020		121	64 %	451,005	72 %
Situation in 2020 of gullies classified as under development in 2004	stable	2	1 %	4318	0.7 %
	unstable	15	8 %	126,262	20.2 %
Total		189		625,927	

### 3.2 Classification of priority gullies

Based on the analysis performed in the inventory, the existing gullies in 2020 were classified according to “local potential for business development”, “attractiveness and marketing”, and “environmental risks”.

#### 3.2.1 Local potential for business development – Class 1

**Fig. 5:**
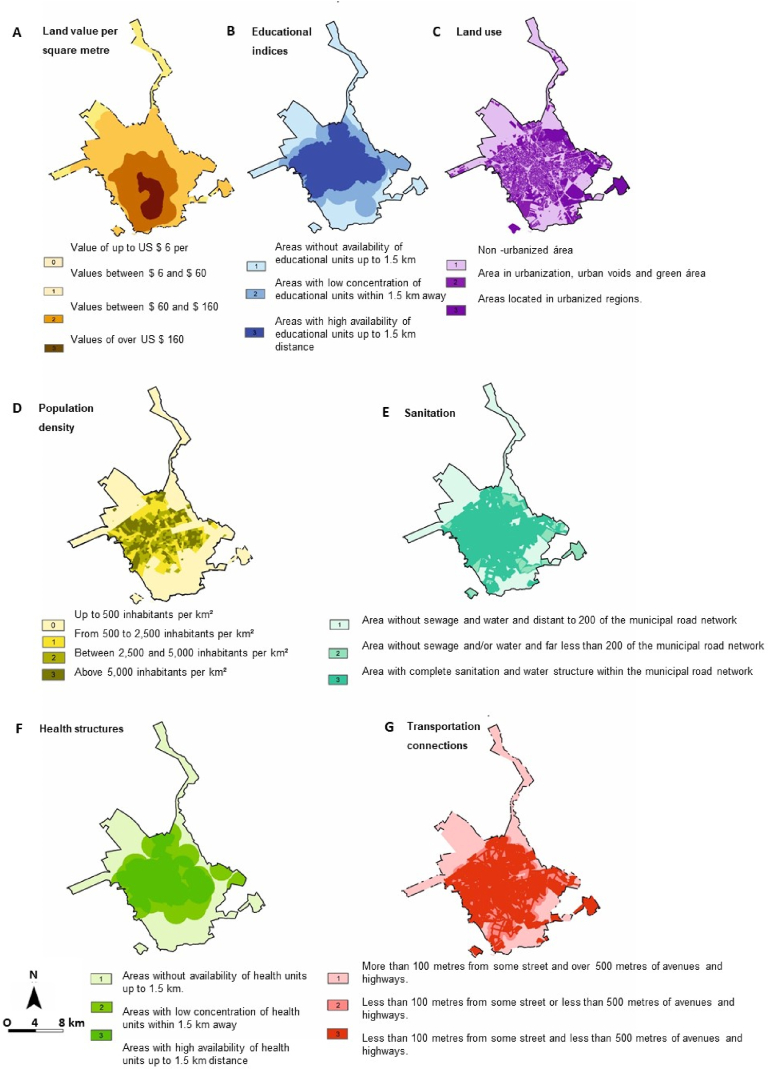
Analysis of the proposed parameters and scores in the work methodology for “Local potential for business development” classification: (A) Land Value per Square Metro; (B) Educational indices; (C) Land use; (D) Population density; (E) Sanitation; (F) Health structure; and (G) Transportation connections. Prepared based on the Master Plan maps [28].

**Fig. 6:**
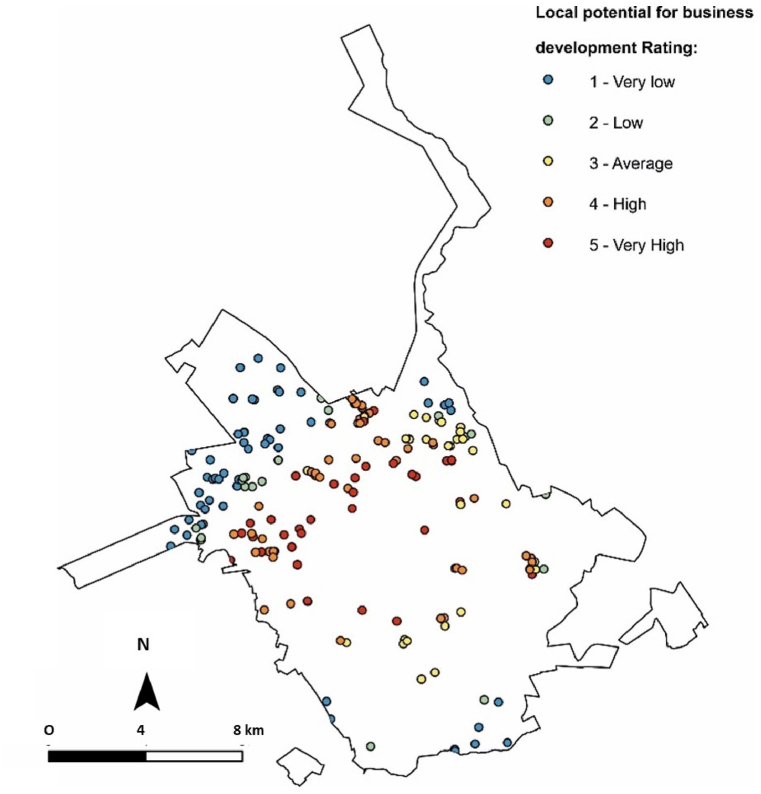
Map indicating the ranking of gullies in relation to the “Local potential for business development”.

Seven parameters were analysed (Fig. 5 and Table 7). Regarding value per square metre, approximately 85 % of gullies occurred in areas with costs between $6 and $60 US dollars. At least 50 % of gullies are in areas with a population density of up to 500 people per hectare. In total, 54 % are in areas of urban expansion with still open spaces. Approximately 50 % of the areas with gullies are in regions close to educational institutions, and 59 % are in regions with a complete sanitation structure, 51 % have good transportation connections. The only parameter that did not obtain any parameter with values greater than 50 % was health structures, the classification showed a distribution of the values between the parameters. The overall classification of this criterion indicated that the areas with the highest scores are in urban expansion zones or in urban open spaces (Fig. 6).

**Table 7:** Relationship between identified gullies and parameters established in the methodology for “Local potential for business development” analysis.

Parameter	Score	Number of gullies	Percentage
Land Value per Square Metro	0	0	0.0 %
	1	162	85.7 %
	2	31	16.4 %
	3	6	3.2 %
Population density	0	96	50.8 %
	1	60	31.7 %
	2	24	12.7 %
	3	9	4.8 %
Educational indices	1	37	19.6 %
	2	57	30.2 %
	3	95	50.3 %
Sanitation	1	67	35.4 %
	2	2	1.1 %
	3	112	59.3 %
Land use	1	103	54.5 %
	2	21	11.1 %
	3	65	34.4 %
Transportation connections	1	66	34.9 %
	2	25	13.2 %
	3	98	51.9 %
Health structure	1	61	32.3 %
	2	56	29.6 %
	3	72	38.1 %

#### 3.2.2 Attractiveness and marketing of the area affected by gullies – Class 2

**Fig. 7:**
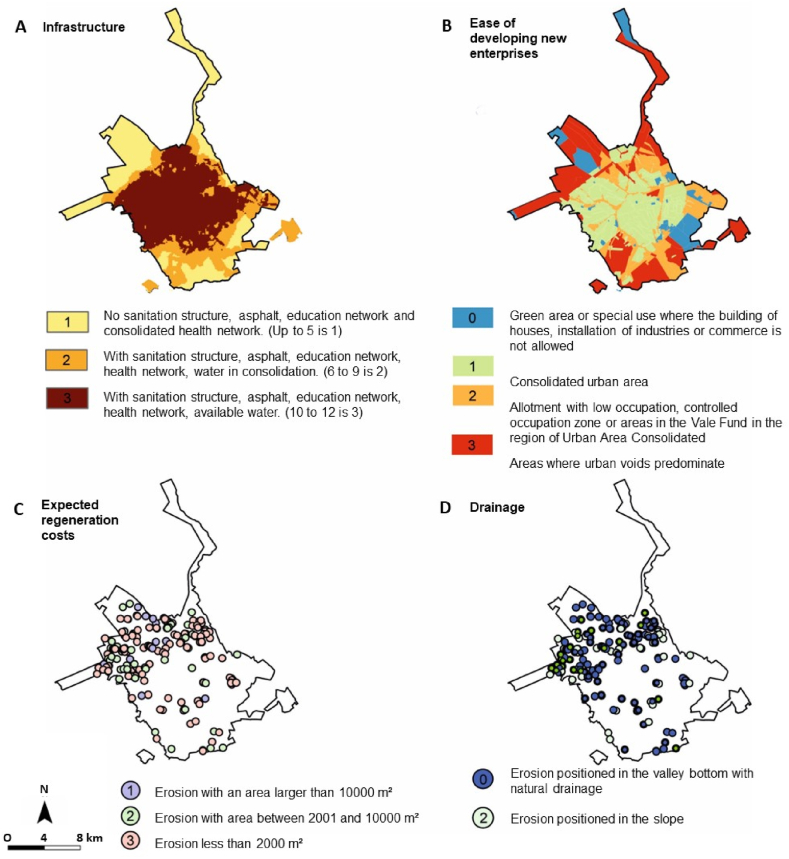
Maps produced for analysis of the proposed parameters and scores in the work methodology for classification of attractiveness and marketing: (A) infrastructure; (B) ease of developing new enterprises; (C) expected regeneration costs; and (D) drainage. Prepared based on the Master Plan maps [28].

The analysis of the attractiveness and marketing of areas affected by the gullies is shown in Fig. 7 and Table 8. Some parameters indicated a correlation greater than 50 % with areas affected by gullies.

**Table 8:** Relationship between identified gullies and parameters established in the methodology for attractiveness and marketing analysis.

Parameters	Score	Number	Percentage
Infrastructure	1	46	24.3 %
	2	43	22.8 %
	3	100	52.9 %
Expected regeneration costs	1	12	6.3 %
	2	53	28 %
	3	124	65.6 %
Ease of developing new enterprises	0	13	6.9 %
	1	39	20.6 %
	2	71	37.6 %
	3	66	34.9 %
Drainage	0	105	55.6 %
	2	84	44.4 %
Possibilities of future uses for residence	0	49	25.9 %
	1	1	0.5 %
	2	0	0 %
	3	139	73.5 %
Possibilities of future uses for trade	0	14	7.4 %
	1	78	41.3 %
	2	57	30.2 %
	3	40	21.2 %
Possibilities of future uses for industry	0	14	7.4 %
	1	115	60.8 %
	2	25	13.2 %
	3	29	15.3 %
	4	6	3.2 %

Most gullies, 52 %, are in areas with good municipal urban structure. Approximately 65 % of gullies affect an area less than 2000 m^2^, and most gullies are in slope areas without contact with natural drainage. The ease of developing new ventures has not indicated any correlation.

**Fig. 8:**
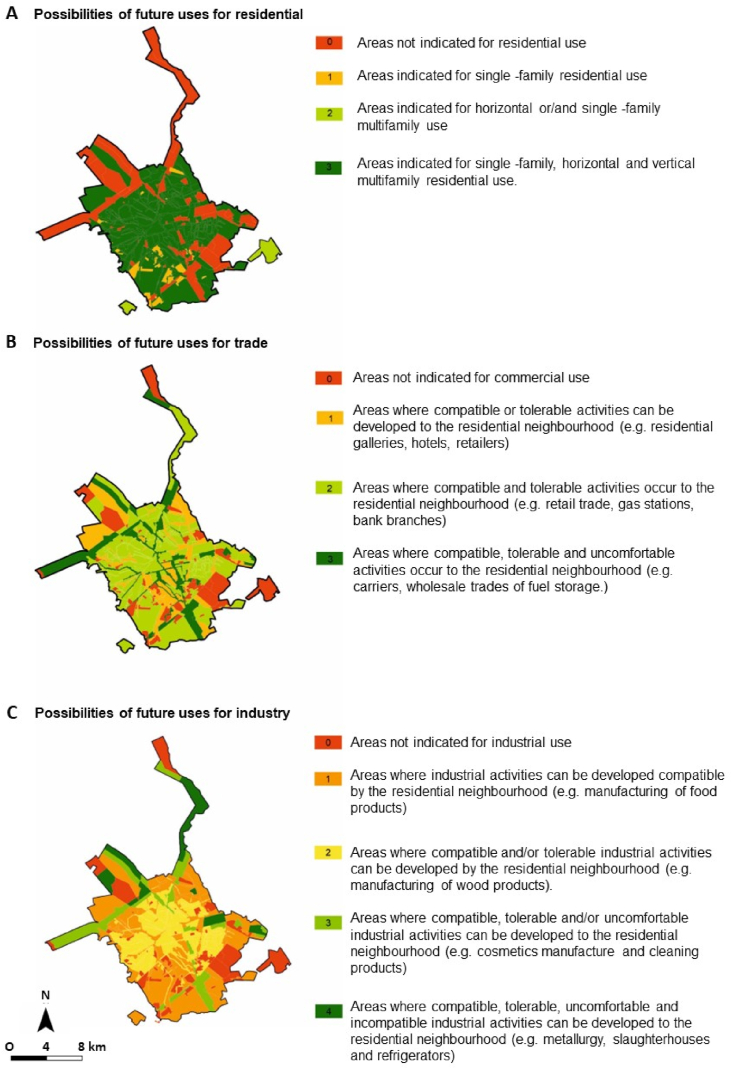
Map with the different possible uses, as indicated in the Municipal Master Plan for each region of the city, considering the (A) possibilities of future uses for residence; (B) possibilities of future uses for trade; and (C) possibilities of future uses for industry [28].

**Fig. 9:**
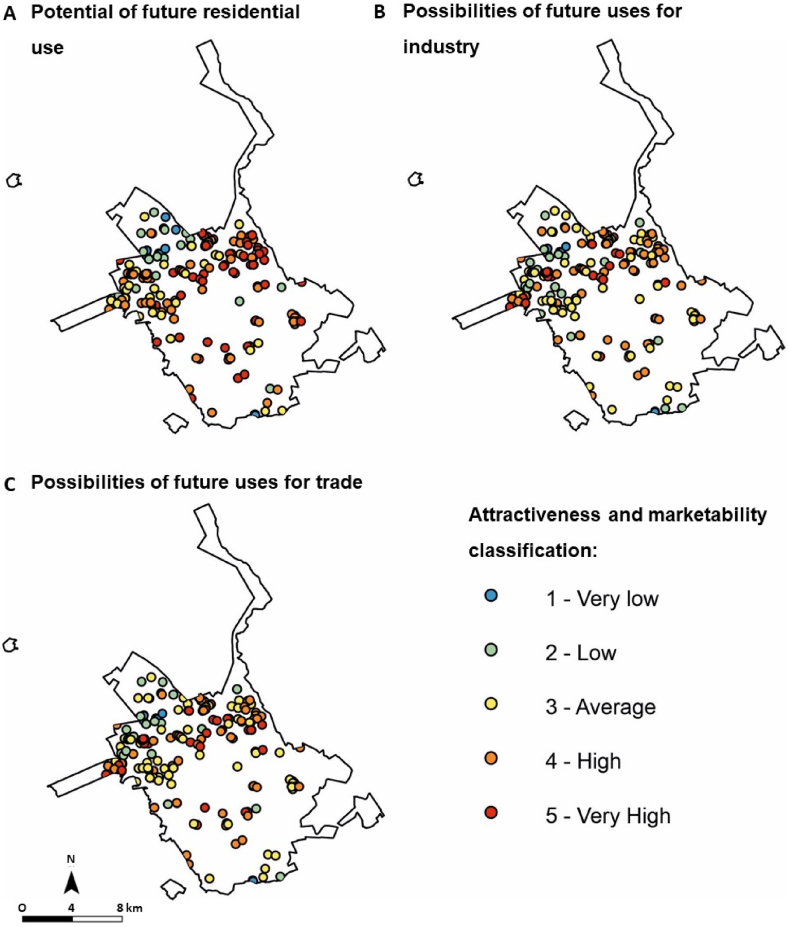
Map indicating the rating of gullies regarding “attractiveness and marketing potential” class, considering the possibilities of (A) residential, (B) industrial and (C) trade use.

Among the possible uses for the areas (Fig. 8), most of them, 73 %, can be used for different residential uses. Use for small business or industrial activities compatible with residential use is indicated in 41 % and 60 %, respectively. As three distinct analyses of use were applied, residential, commercial and industrial, three different rating were also generated (Fig. 9). The result indicates the three different classifications for the “attractiveness and marketing potential” class, according to the existing municipal master plan restrictions for residential, commercial or industrial use.

#### 3.2.3 Environmental risks - Class 3

**Fig. 10:**
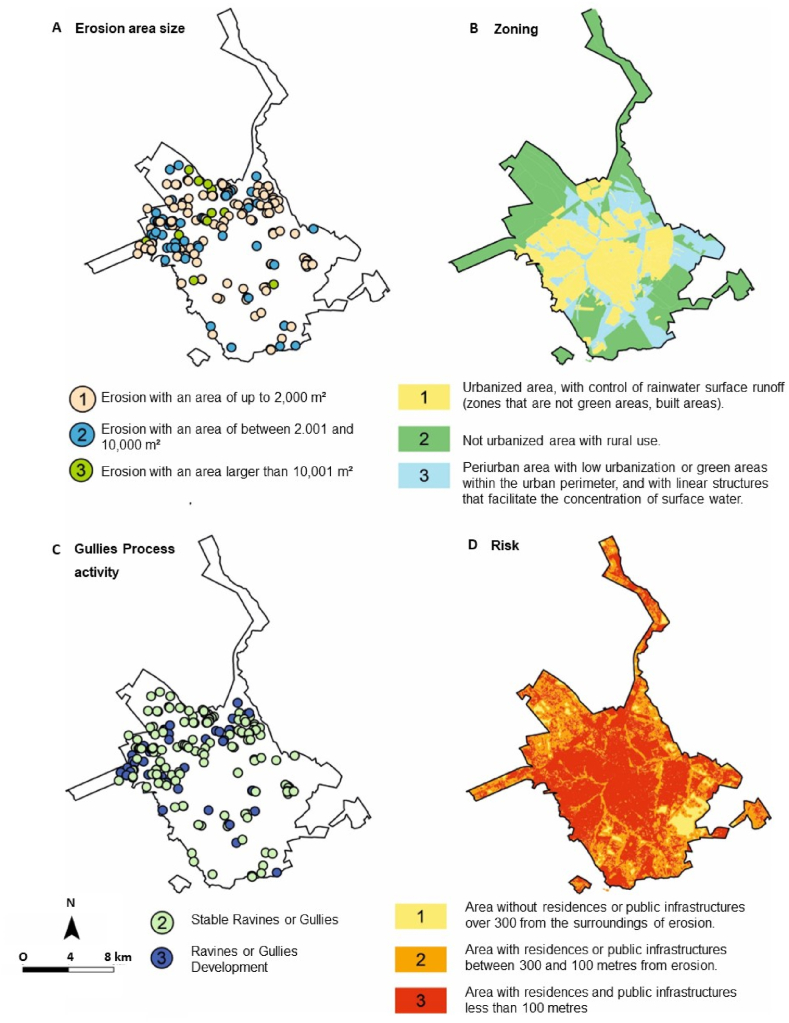
Maps produced for analysis of the proposed parameters and scores in the work methodology for environmental risk classification: (A) gullies area size; (B) zoning; (C) gullies process activity; and (D) risk: proximity with urban structures. Prepared based on the Master Plan maps [28].

Four parameters were analysed (Table 9 and Fig. 10). Environmental risk analyses considered the size of the area of gully; whether gully was active or still developing, urban zoning in relation to urbanized areas, areas with urban voids and nonurbanized areas and green areas, and, in the risk analysis, proximity to urban structures was considered.

**Table 9:** Relationship between identified gullies and parameters established in the methodology for environmental risk analysis.

Parameters	Score	Number	Percentage
Gullies area size	1	124	65.6 %
	2	53	28 %
	3	12	6.3 %
Gullies Process activity	2	139	73.5 %
	3	50	26.5 %
Zoning	1	39	20.6 %
	2	78	41.3 %
	3	72	38.1 %
Risk: Proximity with urban structures	1	0	0 %
	2	95	50.3 %
	3	94	49.7 %

**Fig. 11:**
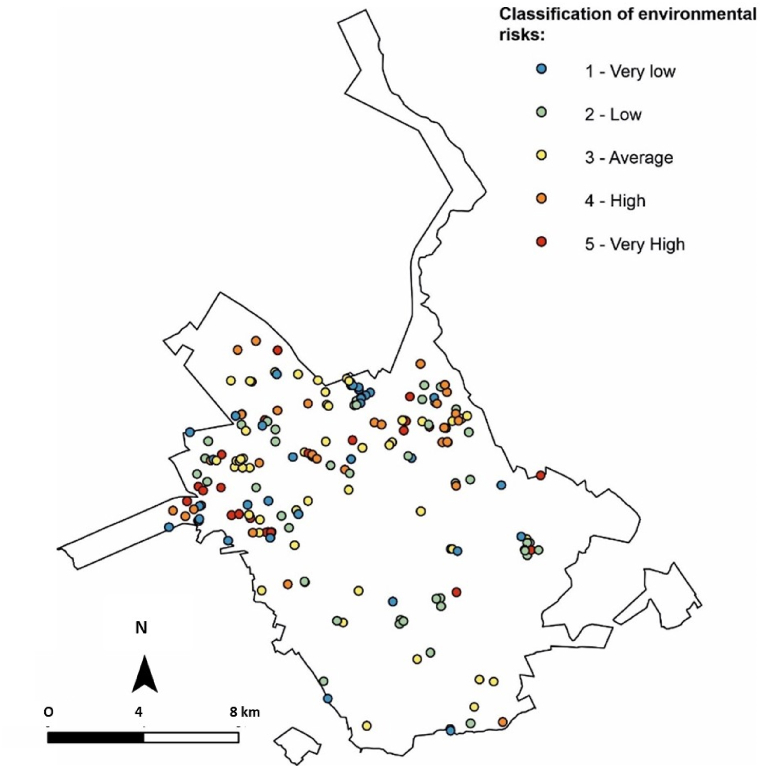
Map indicating the classification of gullies in relation to environmental risks.

Most gullies, 65 %, have an area of less than 2000 m^2^. The analysis indicates a correlation with gully activity, and 73 % of gullies are classified as stable. In the zoning class, there was no strong correlation with any of the classes. All gullies analysed are at least 300 m from some public or private infrastructure. Based on the criteria analysed, gullies were classified in relation to environmental risks (Fig. 11).

### 3.3 Analysis of priority areas

The classification can be analysed individually for each of the 3 classes or analysed in an integrated way. Gullies that have a high rating considering the 3 classes, are cases where, if prioritized, they can at the same time reduce the risk and present economic gains if the area is reintroduced for urban use.

**Fig. 12:**
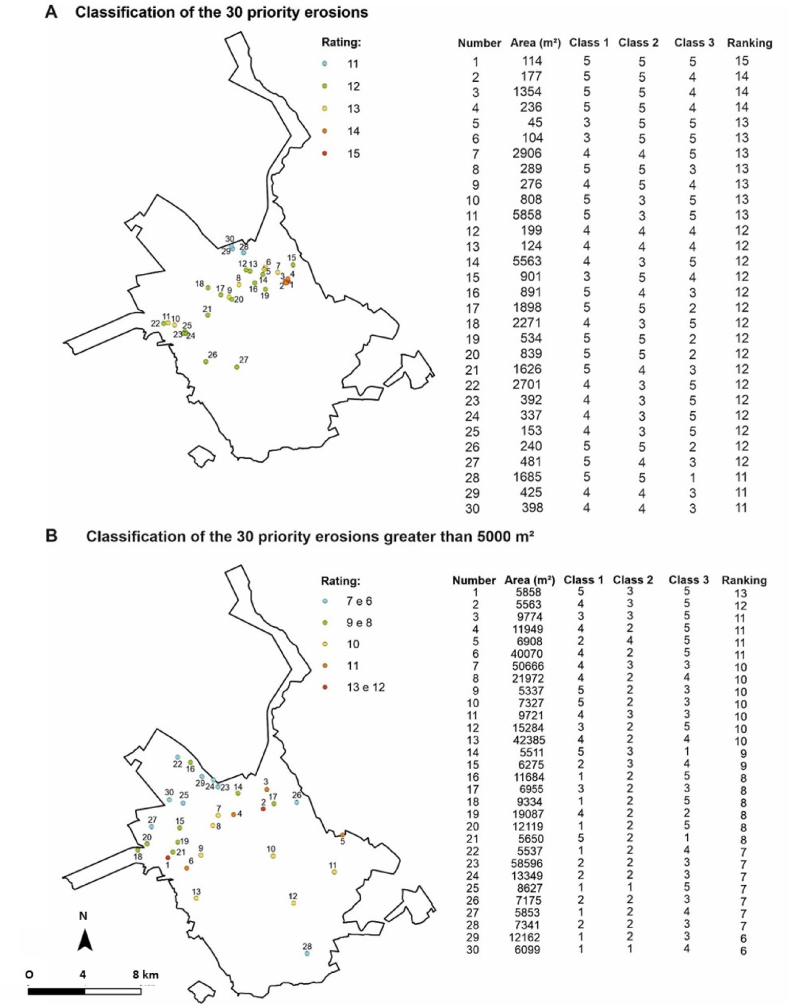
Considering the three classes considered: local potential for business development (Class 1); attractiveness and marketing potential (Class 2); and environmental risks (Class 3), the figure shows the classification of priority gullies: a) map with the 30 best classified gullies; B) map with the 30 gully larger than 5000 m^2^ better classified.

The 30 best classified gullies when adding the rating of the 3 classes analysed are concentrated mostly in urban expansions, northwest of the city (Fig. 12 A). The 7 gullies with the highest potential are concentrated in the Quinta da Bela Olinda region (Fig. 4C and D). The data indicate that this is the area with the highest development potential today and has gully with the highest risk of activities.

When considering the 30 best classified gullies with an area greater than 5000 m^2^ (Fig. 12 B), although there is still a larger concentration of gullies in the northwest sector of the city, well-classified gullies also occur in other sectors, indicating a greater dispersion of erosive processes.

## 4 Discussion

The inventory indicated an increase in the number of gullies between 2004 and 2020 from 175 to 189. The area directly affected by gully channels decreased from 64.1 ha to 62.5 ha. The contrast between the increased number of gullies and the reduced area is the result of the remediation of large channels among the 37 recovered gullies; most small gullies (51 new channels) emerged between 2004 and 2020, indicating that gullies will increase in number, but these gullies still have a smaller size. Although the reduction in the affected area is good news, the existence of a greater number of gullies may indicate a growth trend in the medium term.

Brownfields are found in urban centers and have potential land resources that can be important areas to reduce land scarcity [30]. Brownfield can be areas of old factories, buildings, railways, military areas, but there are also authors who consider mining areas, semi-urban areas and agricultural areas [31,32]. Gullies are generated by natural and anthropogenic factors [33], and in urban areas they are related to inadequate land use [4,8]. In other cities in Brazil and around the world, gullies also occupy significant areas and are a challenge for the development of cities, due to the creation of abandoned areas [4,8,[33], [34], [35]]. Gullies can therefore be classified as a form of Brownfield, as they are generated due to inadequate soil use, making the use of land in areas that could previously be occupied unfeasible, but in the same way as occurs with other Brownfields, if the necessary interventions, these areas can be reinserted for land use.

Although the area directly affected by gully channels is 62.5 ha, the surrounding area indirectly affected can easily be 10 times larger, as the gullies make the use of the surrounding area unfeasible. This demonstrates that gullies represent a problem with very significant dimensions. Reintroducing these spaces for urban use is crucial in city management. Although they are a significant challenge, the existence of large urban areas without occupation is also an opportunity, and the size of brownfields in hectares was a positive parameter for areas to be recovered [36]. The recovery of areas affected by gully also prevents new damage in the vicinity. The inventory indicated that the areas of urban expansion have the largest number of gullies, while in consolidated urban areas, new gullies in most cases are related to inadequate drainage structures.

Regarding the class “local potential for business development”, among the parameters, the strongest correlation was in relation to costs per square metre between $6 and $60, which was one of the lowest scores in the analysis. This indicates a relationship between the existence of gully and the low value of the property. However, this correlation can also indicate an opportunity. Low land prices increase attractiveness for potential investors [17]. However, the data also indicated that most areas affected by gully are in places close to educational institutions and with public sanitation and good transportation calls. Thus, the recovery of areas affected by gullies can be a good opportunity considering the existing public infrastructures in the surroundings. In the long run, the trend is that the areas affected by gullies that are not considered today for new enterprises become attractive due to their location in the city. Local potential analyses developed in this work can accelerate this process due to the creation of indicators that demonstrate the competitive differences of each area.

The class “attractiveness and marketing” showed that most gullies are positioned in places with good urban infrastructure and have a scar with an area of less than 2000 m^2^. According to Ref. [37], small gullies have not only lower costs for regeneration but also less technical complexity than large gullies. Regarding the ease of development of new enterprises, a strong correlation with any proposed class was not indicated. However, large urban voids may provide excellent opportunities for large enterprises, which helps to cover eventual remediation costs [36].

The analysis of attractiveness and marketing allows us to relate gullies to the possibilities provided for in the city's master plan. This is interesting to identify areas most suitable for specific use according to municipal standards. Residential use was the most suitable in 73 % of cases, but small industries and trades were also possible in 60 % and 41 % of areas, respectively. If enterprises seek the construction of large industries and trades, the map produced also allows an easy view of the areas possible to develop this activity.Low potential areas for these three uses (residential, industries and trades) can be used as green space or left as brownfield, which does not involve the construction of infrastructure [38]. Alternative uses may be connected to geotourism and education programs [39]. Municipal public policies and tax incentives can assist in more sustainable land use, allowing the best option to be taken for the urban community [17].

Risk analysis has indicated that all existing gullies in the area are at least 300 m from some public or private infrastructure. This indicates the need for monitoring all gullies, as in some cases, these gullies may have a rapid evolution, and if mediation measures are not performed, they can destroy infrastructures and even cause fatalities. Most gullies are less than 2000 m^2^ and classified as stable, so the reactivation of these channels or the rapid growth of smaller channels may occur when soil use and occupation change or extreme weather events occur.The three priority categories can be analysed separately according to the user's interest; for example, if the interest is to find areas with good local potential, only class 1 should be considered, but if the goal is to analyse or monitor the evolution of the gully risk, class 3 will indicate priority gullies that may cause significant economic or environmental damage.The final priority analysis indicated that the area of Quinta da Bela Olinda has better classified gullies, considering the sum of the three priority categories. This reinforces the analysis performed by Ref. [11], who estimated that approximately 80.7 ha in that region are affected due to the existence of gullies. The environmental and economic impacts calculated by the authors demonstrate that losses exceed $76.4 Million over the last few decades. The area was planned for residential use, but the development of gullies over the last three decades made it impossible to use the site. Reintegrating this area into the urban environment represents a good opportunity for municipal entrepreneurs and managers, but this requires creating marketing strategies to convince local actors. The creation of databases that bring together information on the techniques used to regenerate the areas, existing costs and monitoring measures applied may represent a risk reduction factor and improve agility in decision-making and application of resources more assertively.

The analysis carried out by applying the TIMBRE methodology allows us to visualize which areas affected by gullies have the greatest potential to return them to use. The classification can indicate among hundreds of gullies which of them are in areas with high potential for urban use. After the classification carried out by the methodology, it is necessary to carry out on-site analyses of the priority gullies. Geomorphology (slope, drainage basin size, strand shape), Geology (rock type, aquifer characteristics), soil (cohesion, permeability, erodibility, soil thickness) and other factors such as characteristic of vegetation and anthropogenic changes in land use, can be important conditions in the gullies analysis, to view details related to any technical difficulties existing in the area [[2], [3], [4],^12^,^13^].

The inventory demonstrated that several areas were reintroduced into the urban environment in the municipality of Bauru during the time interval analysed, thus indicating that the reintroduction of gully brownfields is possible. However, the creation of urban green areas in places affected by gullies is also an alternative. The TIMBRE methodology applied to gully classification can be an important ally for managers' decision-making.Although the data collected in this study provide important information on the areas with the highest potential, it is necessary to consult the population and local managers to build consensus and establish priorities for use for the affected areas. This will also depend on land ownership (public vs. private land).

To convince stakeholders, dialogue strategies must be developed [40]. identified five groups interested in brownfield regeneration: site owners, authorities, neighbours and others interested in the problems related to the area, service providers, and scientific community. These same authors propose a participatory methodology consisting of five phases, which involves (i) planning and preparatory work, (ii) mapping of stakeholders, (iii) development of activities such as workshops and lectures to foster engagement, (iv) application of a devolutionary questionnaire and (V) feedback for each of the stakeholders involved.

The establishment of a governance strategy through policies is another factor that can help in the regeneration of Brownfields, as the time required for complete implementation of a municipal or even project strategy may be longer than the period of managers' electoral mandates [41]. The classification presented here provides an important indicator for gully brownfield management, just as it is already done with other types of brownfield portfolios. To identify the variables that are most relevant for area recovery. The construction of databases assisting the analysis of territorial evolution can be an important metric to make decision-making increasingly assertive.

Application of this method to other places is possible provided it is adjusted to local data. The development of decision support systems (DSS) for gullies management in urban areas can help solve the complexity involved in the areas, as proposed for contaminated areas [42]. Tools also help identify and manage the specific opportunities and risks of each remediation project [43].

The methodology can also help other types of analysis, for example, for insurance companies to establish the prices for lands or buildings according to the risk scores. It can also help investors to define the risks that exist for investments in areas affected by gullies. Thus, the proposed method's main strength is the ability to visualize and classify many gullies in a wide area. However, this regional analysis is based on simplifications, which is a limitation of the work. Therefore, after choosing the priority gullies, it is necessary to detail the characteristics related to the physical and social environment of each of them.

## 5 Conclusions

This work demonstrated that the TIMBRE method can be successfully adapted for the analysis brownfields caused by gullies. This adaptation allows for an investigation of which are the priority areas for gully development, facilitating decision-making by managers, communities and investors in cities where this problem is relevant. The use of this type of analysis can help in the reintroduction of important idle spaces in cities in developing countries, where the disorderly growth of urban areas has favoured the creation of gully-derived brownfields.

The inventory performed by this work showed an increase in the number of gullies in the urban perimeter; however, the area affected by gully scars was reduced by approximately 2 ha. The recovery of areas affected by gully was mainly due to urban structure works and residential construction. Brownfields are concentrated in peripheral regions and in the urban growth zone. However, many of these areas have a good urban infrastructure and several services nearby. Creating pathways that facilitate reintroduction reduces the cost of development of new housing, industries or trade.

The adaptation of the gullies analysis methodology is a first step, but further research needs to be performed to create more complete databases that allow sharing knowledge between different cities and countries that have gully-derived brownfield problems. The construction of this type of database allows for an analysis of good practices or even the registration and study of unsuccessful cases.

## CRediT authorship contribution statement

**Caiubi Emanuel Souza Kuhn:** Writing – original draft, Methodology, Formal analysis, Data curation, Conceptualization. **Fábio Augusto Gomes Vieira Reis:** Writing – review & editing, Conceptualization. **Flávia Regina Pereira Santos:** Writing – review & editing. **Christiane Zarfl:** Writing – review & editing. **Peter Grathwohl:** Writing – review & editing. **Victor Cabral:** Writing – review & editing.

## Declaration of competing interest

The authors declare that they have no known competing financial interests or personal relationships that could have appeared to influence the work reported in this paper.
